# A portable, programmable, multichannel stimulator with high compliance voltage for noninvasive neural stimulation of motor and sensory nerves in humans

**DOI:** 10.1038/s41598-023-30545-8

**Published:** 2023-03-01

**Authors:** Marshall A. Trout, Abigail T. Harrison, Mark R. Brinton, Jacob A. George

**Affiliations:** 1 Department of Electrical and Computer Engineering, University of Utah, Salt Lake City, USA; 2 Department of Biomedical Engineering, University of Utah, Salt Lake City, USA; 3 Department of Engineering and Physics, Elizabethtown College, Elizabethtown, USA; 4 Department of Physical Medicine and Rehabilitation, University of Utah, Salt Lake City, USA; 5 Department of Mechanical Engineering, University of Utah, Salt Lake City, USA

**Keywords:** Health care, Medical research, Biomedical engineering, Electrical and electronic engineering

## Abstract

Most neural stimulators do not have a high enough compliance voltage to pass current through the skin. The few stimulators that meet the high compliance voltage necessary for transcutaneous stimulation are typically large benchtop units that are not portable, and the stimulation waveforms cannot be readily customized. To address this, we present the design and validation of a portable, programmable, multichannel, noninvasive neural stimulator that can generate three custom bipolar waveforms at ± 150 V with microsecond temporal resolution. The design is low-cost, open-source, and validated on the benchtop and with a healthy population to demonstrate its functionality for sensory and motor stimulation. Sensory stimulation included electrocutaneous stimulation targeting cutaneous mechanoreceptors at the surface of the skin and transcutaneous nerve stimulation targeting the median nerve at the wrist. Both electrocutaneous stimulation on the hand and transcutaneous stimulation at the wrist can elicit isolated tactile percepts on the hand but changes in pulse frequency are more discriminable for electrocutaneous stimulation. Also, neuromuscular electrical stimulation of the *flexor digiti profundus* is evoked by applying electrical stimulation directly above the muscle in the forearm and to the *median* and *ulnar* nerves in the upper arm. Muscle and nerve stimulation evoked similar grip forces and force rise times, but nerve stimulation had a significantly slower fatigue rate. The development and validation of this noninvasive stimulator and direct comparison of common sensory and motor stimulation targets in a human population constitute an important step towards more widespread use and accessibility of neural stimulation for education and research.

## Introduction

Electrical stimulation of the peripheral nerves can be used to activate motor or sensory fibers. Activating motor fibers or the muscles they innervate produces muscle contractions that can be used to reanimate paralyzed limbs for assistive^1,2^ or rehabilitative purposes^3–7^. Activating mechanoreceptive and proprioceptive sensory fibers produces discriminable percepts that can be used for real-time haptic feedback for assistive technology^8–12^ or for virtual or augmented reality interfaces^13,14^.

To activate motor fibers, stimulation is traditionally applied directly above the muscle belly to cause the muscle nearest the electrode to contract. More recently, stimulation has been applied to the proximal nerve truck to activate motor units that innervate distal and deeper muscles^15–19^. Sensory fibers can be activated through either electrocutaneous or transcutaneous stimulation. Electrocutaneous stimulation (also known as electrotactile stimulation) primarily activates the distal end of mechanoreceptors at the surface of the skin where they contact an electrode, which in turn evokes a percept at the site of the electrode. Transcutaneous stimulation (also referred to as proximal nerve stimulation) passes current deeper through the skin to activate sensory fibers within a nerve bundle, which in turn evokes a more distal percept at the receptive field of the activated mechanoreceptor nerve fiber.

All four of these noninvasive stimulation approaches require a high compliance voltage to drive current through the high impedance of the skin. Each stimulation approach also requires unique stimulation parameters (e.g., pulse frequency and current). Bipolar stimulation and rapidly interleaved multichannel stimulation are commonly techniques used to steer the electrical current to precisely target underlying neural structures and activate multiple fibers simultaneously^1,20^. Thus, an ideal noninvasive neural stimulator requires high compliance voltage, programmable stimulation parameters, multiple channels, and the ability to rapidly switch between multiple channels.

Most stimulators on the market are intended for invasive applications and do not have high enough compliance voltage to pass current through the skin^21–26^. Even commercial transcutaneous electrical nerve stimulation (TENS) units only have a compliance voltage of 30 V, which is far below the 300 V necessary to pass current through the skin with small, localized electrodes. TENS units are also typically limited to a single channel, and the waveforms cannot be readily customized. The few commercial stimulators that have compliance voltages and programmable stimulation parameters are expensive, stationary, desktop units with limited channels^26–30^. Custom configurations used in research often incorporate a mechanical switch matrix to multiplex one channel to multiple electrode sites^1,15^; however, the switching speed of mechanical switch matrices is slow and prohibits rapid interleaving of stimulation for simultaneously activating multiple neural structures^31^.

We present a portable, programmable, multichannel neural stimulator designed with 300 V compliance for noninvasive stimulation. The noninvasive stimulator is also low-cost and open-source. The efficacy of the stimulator is validated with human participants by performing novel, direct comparisons of noninvasive (a) electrocutaneous and transcutaneous sensory stimulation for sensory feedback, and (b) muscle and nerve stimulation for motor activation. Our experimental validation provides new insight into the relative merits of different stimulation targets for both motor and sensory applications. Furthermore, as demonstrated by the experiments herein, the development of this noninvasive stimulator enables a variety of different research studies and may enable more widespread use of noninvasive stimulation for educational and research purposes.

## Materials and methods

### High-voltage biphasic current-controlled pulses

To deliver safe, efficacious stimulation for long periods of time, a neural stimulator needs to be able to deliver current-controlled, biphasic pulses. Current-controlled stimulation allows the user to directly control the charge density of each waveform, irrespective of impedance at a given site. Adverse skin reactions and discomfort can occur if noninvasive stimulation is monophasic or has an excessive charge density^32,33^. For small, dry electrodes, the impedance at the electrode–skin interface can be large (around 25 kΩ for a 0.44 cm^2^ electrode), which requires a stimulator with compliance voltages of at least ± 150 V in order to drive currents of 6 mA (Table 1) through the electrode–skin interface^8^.

**Table 1:** High-voltage noninvasive stimulator specifications.

Electrode impedance	25 kΩ
Compliance voltage	± 150 V
Stimulation current	6 mA
Time resolution	5 µs
Stimulation frequency range	1–250 Hz
Rise time	~ 2 µs
Update frequency	~ 90 kHz

**Figure 1:**
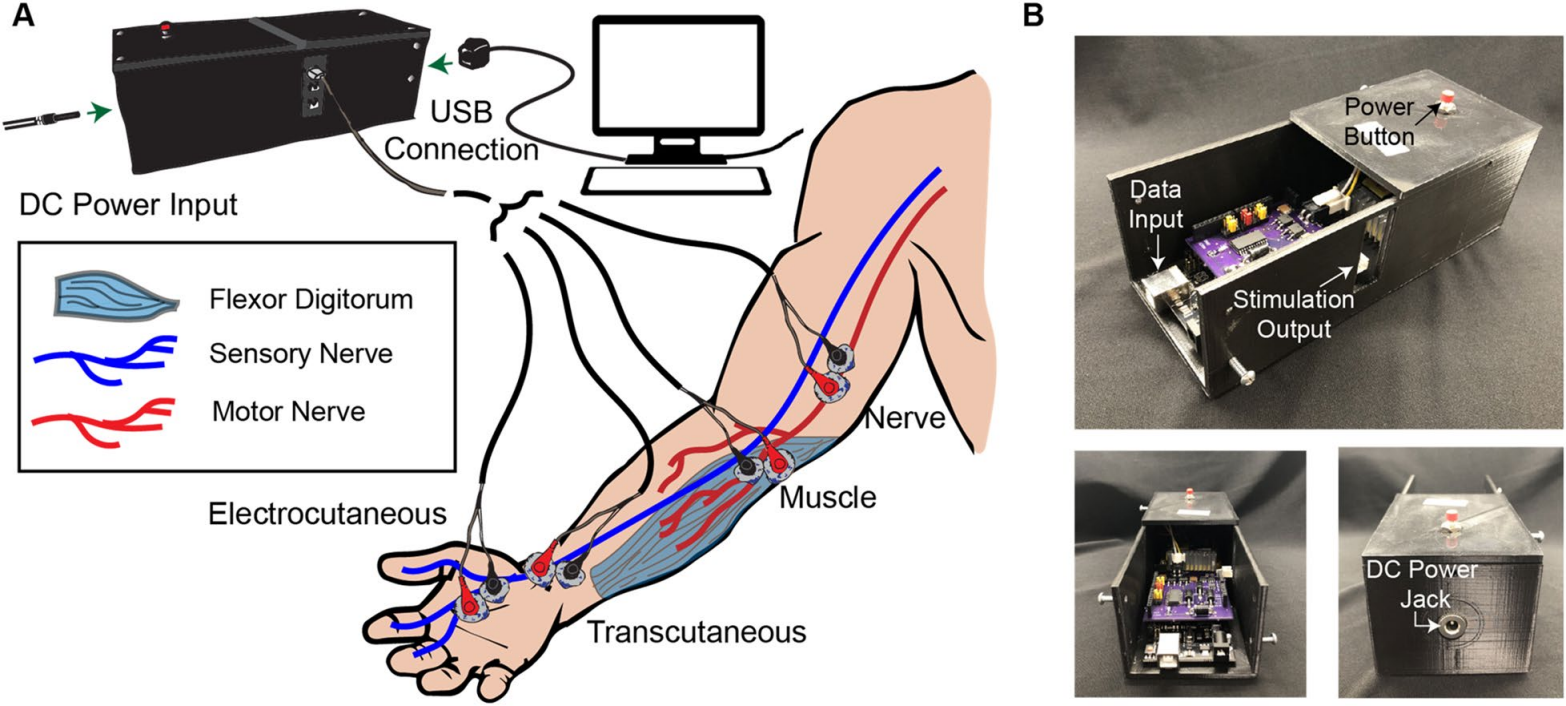
Overview of neural stimulator. (**A**) The stimulator is powered with a 12-V DC barrel connector or battery and communicates with external software using a USB-B connection. The stimulator can be used to noninvasively activate sensory afferent fibers via electrocutaneous applied directly to the sensation site and transcutaneous stimulation applied to the nerve before it innervates the sensory organ. The stimulator also noninvasively activates motor efferent fibers via direct application to the muscle or indirectly to the nerve. (**B**) The stimulator consists of a microcontroller, the stimulation driver, and a DC-DC boost converter encased in a 3D-printed housing. The power to the DC-DC boost converter is connected to a push button that acts as an emergency stop. The stimulator circuitry is designed to stack with the Arduino Uno form factor such that it can be combined with other educational tools built around the Uno form factor.

**Figure 2:**
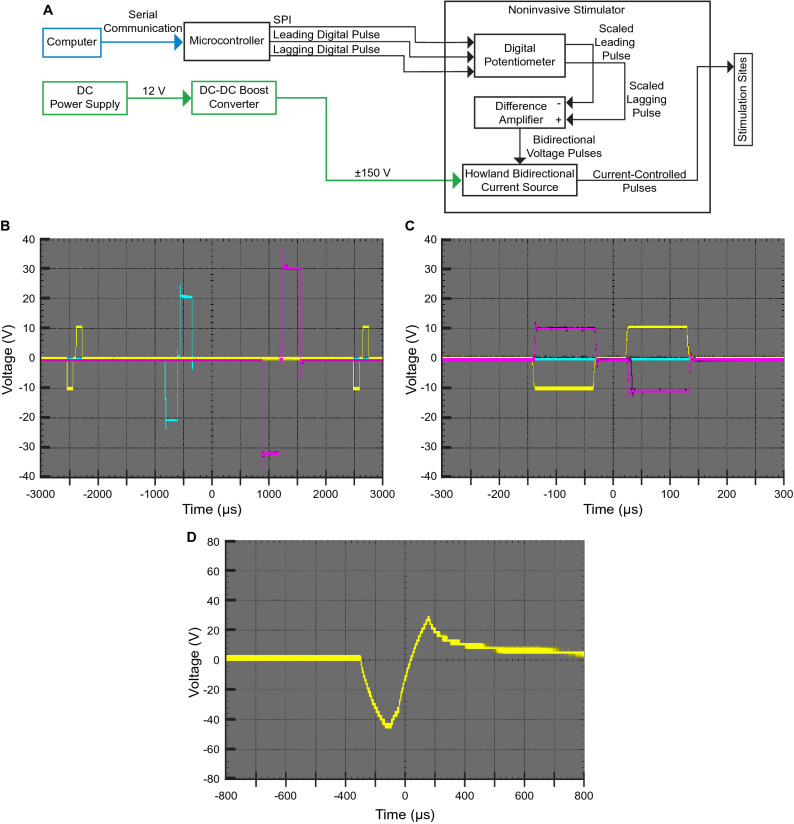
(**A**) The stimulator can consist of three stimulator boards stacked on top of an Arduino Uno microcontroller. The stimulus is defined by commands written via serial communication. The digital pulses are scaled by the digital potentiometer which is controlled via SPI. A differential amplifier inverts one of the pulses and combines it with the positive pulse. The voltage pulses are the input to the Howland, bidirectional current source. The Howland current source is powered by a DC-DC boost converter that is in turn powered by a 12-V power source. (**B**) The stimulator produces a series of interleaved biphasic pulses of varying amplitudes, driving current between that channel and a common ground electrode. The yellow line represents channel one, the blue line represents channel two, and the pink line represents channel three. Vertical gradations indicate 10 V and the horizontal gradations indicate 500 µs. (**C**) The stimulator can also produce bipolar stimulation across multiple channels. One channel provides a cathodic pulse while another channel provides an anodic pulse simultaneously. Vertical gradations indicate 10 V and the horizontal gradations indicate 50 µs. The overshoot seen in some of the waveforms is common to this Howland current source design. The charge associated the short overshoot is negligible. The trapezoidal shape of the square waves is cause by the slew rate of the op-amps used in the design. However, this linear region is small enough to not effect operation. (**D**) The noninvasive stimulator delivering a 3-mA 300-µs cathodic-first biphasic waveform with a 50-µs interphase interval through the skin via adhesive gel electrodes. The voltage across the electrodes was measured using an oscilloscope. Vertical gradations indicate 20 V and the horizontal gradations indicate 200 µs. A compliance voltage of approximately 46 V is observed when driving 3 mA. Higher compliance voltages (up to 150 V) are necessary to drive higher currents through the skin.

The noninvasive stimulator design is based on the Howland bidirectional current supply (Fig. 2A & Supp. Fig. 1) seen in^34^. A development board (Uno, Arduino LLC, Boston, MA) is used to control the noninvasive stimulator. Two square waves are sequentially generated using two different digital output pins on the microcontroller for the cathodic and anodic portions of the waveform. The stimulator’s amplitudes are individually controlled via serial-peripheral interface (SPI) commands that are written to a digital potentiometer (AD5204BRZ10, Analog Devices, Norwood, MA). The two square waves are generated by the microcontroller’s digital output pins using the custom firmware. The square waves are connected to the digital potentiometer such that the wiper voltage is the scaled output (Fig. 2A & Supp. Fig. 1). A difference amplifier (INA149AIDR, Texas Instruments, Austin, TX) is then used to invert the cathodic phase and combine it with the anodic phase. The combined waveform is then used as the input to the Howland bidirectional current supply (Fig. 2A & Supp. Fig. 1). A DC boost converter supplies ± 150 V to each Howland current supply to drive the current. An overview of the system can be found in Fig. 1, and a block diagram of the noninvasive stimulator can be found in Fig. 2A. The electronic characteristics of the system can be found in Table 1.

### Programmable waveforms

The magnitude and type of response elicited by electrical stimulation is dependent on the amplitude, frequency, and pulse width of the stimulus waveform^8,15,16,35^. The ability to adjust the amplitude, frequency, and pulse width in real time is necessary for closed-loop applications. Being able to adjust stimulation parameters in real-time is also useful for characterizing evoked movements or percepts^8,20^. For the system to be used with closed-loop applications, stimulation parameters must be controllable online in real-time^6,11^.

The microcontroller is programmed with firmware that defines stimulation. Both the digital potentiometer that controls the amplitude of the stimulus and the digital input/output pins that determine the active phase can be set up by each individual user by defining the stimulation parameters in the microcontroller’s software and uploading the code to the microcontroller. The development board receives commands over a serial communication port to allow real-time adjustments of the stimulation parameters. For the applications presented in this work, the firmware was designed to generate two square pulses offset with an interphase interval of 50 µs. The pulse width and frequency of the square pulses were set using values received from serial communication. The amplitude of the square waves was set by varying the resistance of the digital potentiometer using values received from serial communication. Stimulation updates are limited by the loop speed of the script running on the microcontroller.

For the sensory and motor experiments presented herein, custom software interfaces were developed to control the noninvasive stimulator in real-time using LabVIEW (version 20.0.1, National Instruments, Austin, TX) and MATLAB (version 2020b, Mathworks Inc., Natick, MA). Serial communications commands were sent using MATLAB to the stimulator using a USB cable that were then interpreted by custom firmware on the microcontroller.

### Multichannel

The nerve fibers activated by noninvasive nerve stimulation are dependent on the location the stimulus is applied^1,15,18,35,36^. Multiple stimulation channels can be connected to distinct electrodes to provide a programmatic way to switch the location of the stimulus. For motor stimulation, multiple channels allow simultaneous and interleaved activation of different muscle groups^1,37^. Similarly, for sensory stimulation, multiple channels allow users to combine percepts and create a more enriched sensory experience^9,10^. Having multiple channels also creates the potential to utilize current steering to activate fibers that would be otherwise inaccessible^1^. A stimulator should have at least two channels to target multiple sites at once.

The noninvasive stimulator is designed to stack on top of an Arduino Uno development board. Shunts are used to select which pins of the microcontroller are used to control each individual stimulation channel. This allows the SPI and waveforms pins of the microcontroller to only be connected to a single stimulator. Each stimulator requires three digital output pins, so up to three stimulator boards can be stacked on a single Arduino Uno development board. Multiple development boards can be used within a single software framework to expand the total number of channels beyond three.

### Low cost and accessibility

A barrier to entry in electrophysiological research is the cost of stimulation systems. Stimulators capable of passing current through the skin vary in cost from $1,182.00 (DS3, , Digitimer, Hertfordshire, UK) to $10,341.15 (DS8R, Digitimer, Hertfordshire, UK)^29,30^. An accessible stimulator would open the field for increased research and education. To be comparable to other commercially-available neuroscience educational tools, the total cost of a single-channel stimulator should be less than $200^38^.

The noninvasive stimulator board consists of a single printed-circuit board built around the Arduino Uno microcontroller (Supp. Fig. 2), which is globally available and costs $23.00^39^. The dimensions of the stimulator board are 59 mm by 56 mm. The Uno shield form factor was chosen for the noninvasive stimulator board due to the Uno’s low-cost and wide availability. The Uno shield form factor lends itself well to educational purposes as the noninvasive stimulator board can electrically and mechanically interface with a Uno development board by stacking the boards on top of one another using the aligned header pins. The stimulator board can also easily stack on top of other neuroscience educational tools in the Uno shield form factor^38^. Combining a microcontroller with a digital potentiometer allows the user to generate custom waveforms without buying external hardware, such as a waveform generator (Fig. 2A). The total cost of parts for each stimulator boards for a single channel comes to $58.92. When adding in the cost of the power supply, DC-DC boost converter, and microcontroller, a single-channel noninvasive stimulator costs approximately $104.84, and a 3-channel system costs $222.68. The itemized cost for a system can be found in Table 2, and a functional comparison to similar stimulation systems can be found in Table 3. The noninvasive stimulator design is available under an open-source license for other researchers at https://github.com/utahneurorobotics/u-of-u-noninvasive-stimulator. We have also provided the circuit schematic and board design in Supplementary Figs. 1 and 2, respectively.

**Table 2:** High-voltage 3-channel stimulator parts cost.

Part	Cost/Part	Quantity	Total cost
Arduino uno	$23.00	1	$23.00
Stimulator board	$58.92	3	$176.76
12 V DC power supply	$12.99	1	$12.99
400 V DC boost converter	$9.93	1	$9.93
Total cost for a 3-channel stimulator:			$222.68

**Table 3:** Comparison of the qualities of various noninvasive stimulators.

Stimulator	Compliance voltage	Current-controlled biphasic waveform	Controlled via PC	Arbitrary waveform	Multi-Channel	Mobile/battery powered	Waveform generator included	Validated in humans	Single-channel cost
Our Stimulator	± 150 V	✔	✔	✔	✔	✔	✔	✔	$104.84
Cornman et al	± 150 V	✔	✔	✔	✔	✔	x	x	$30
Poletto & Van Doren	800 V	x	✔	x	✔	N/A	x	✔	> $1300
Schaning & Kaczmarek	± 600 V	x	✔	x	✔	x	x	✔	> $700
BIOPAC STIMISOLA	± 200 V	✔	✔	✔	x	x	x	✔	$1,695*
STG4002	± 120 V	✔	✔	✔	✔	x	✔	✔	$4,773*
DS3	90 V	x	✔	x	x	✔	✔	✔	$1,182*
DS8R	± 200 V	x	✔	x	x	x	✔	✔	$10,341*
Backyard brains HHI	± 95 V	x	x	x	x	✔	✔	✔	$299*,^†^
TENS 7000	50 V	x	x	x	x	✔	✔	✔	$30*

*These commercial system’s prices include manufacturing, safety certification, and other associated costs, as opposed to just parts.

^†^The Backyard Brains Human–Human Interface also includes hardware to record electromyography signals and other supporting hardware which is reflected in the cost.

### Safety

Although the noninvasive stimulator is not intended for medical use, safety is always a paramount concern for electrical stimulation. The stimulator should be simple to disconnect from the power source in the event of discomfort or pain. To protect from device failure due to a short, the stimulator also needs to be able to be isolated from an AC power source.

An emergency stop is situated on the top of the plastic housing between the DC power jack and the DC boost converter such that the device can be quickly disconnected from its power source. The device can be powered from either a battery or the wall outlet through the DC power jack. Powering through a battery will protect the participant in the event of a short circuit developing. An isolator should be placed between the AC power source and the DC power supply if the device is powered through electrical mains. These safety considerations increase the safety of the stimulator in research and educational settings. Nevertheless, it is important to recognize that the stimulator is not a medical device intended to treat medical conditions. There is no explicit feedback control integrated into the circuitry to protect from component failure, and as such, the stimulator should only be used in monitored lab settings for research or educational purposes under the supervision of trained professionals^40–42^.

### Verification of stimulator design

We verified the functionality of the noninvasive stimulator by interleaving stimulus across all three channels and by stimulating two of the three channels in a bipolar electrode configuration (such that one channel was cathodic while the other was anodic). In the interleaved configuration, all channels were operated at 200 Hz. The amplitudes of channels one, two, and three were set to 1, 2, and 3 mA, respectively. The pulse widths of channels one, two, and three were set to 100, 200, and 300 µs, respectively. During the validation of the bipolar electrode configuration, the amplitude of each waveform was set to 1 mA with a pulse width of 100 µs. A 50-µs interpulse interval was used for all waveforms. The loop speed of the microcontroller was timed while three channels were stimulating simultaneously. The output of each channel was connected to a 10 kΩ resistor in all scenarios, and voltage was observed using an oscilloscope (Rigol, Beijing, China). The 10 kΩ resistor was used to test the output of the system in a simple non-capacitive scenario.

The noninvasive stimulator’s output was then validated for use with the human body. Two 716-mm2 (30 mm diameter) adhesive electrodes (Series 530, Cardinal Health, Dublin, Ireland) were placed on the forearm of a healthy participant. A 30 Hz biphasic pulse with an amplitude of 3 mA and a pulse width of 200 µs was then applied to the participant’s forearm using a single channel of the noninvasive stimulator. The resulting voltage across the electrodes was then measured using an oscilloscope.

### Electrocutaneous and transcutaneous sensory stimulation

A total of 14 human participants were recruited in this study. Electrocutaneous and transcutaneous stimulation were both performed with seven participants. Informed consent and experimental protocols were carried out in accordance with the University of Utah Institutional Review Board (IRB 00110994).

**Figure 3:**
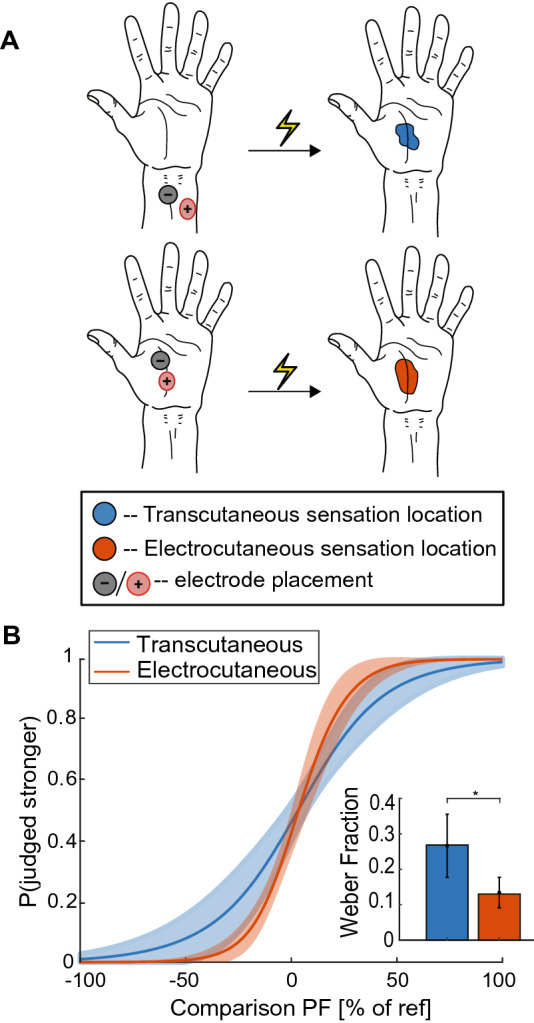
Comparison of transcutaneous stimulation (n = 6, blue) and electrocutaneous stimulation (n = 7, orange). (**A**) Placement of electrodes and the sensory field of evoked percepts. Both forms of stimulation had similar sensory fields despite different electrode locations. (**B**) Psychometric functions relating intensity discrimination performance to changes in pulse frequency. Discrimination performance is given as the percentage of test stimuli identified stronger. Pulse frequency (PF) is reported as a percentage of the reference pulse frequency. Inset shows the Weber fraction for each form of stimulation as mean ± standard error of the mean. Electrocutaneous stimulation (red) had a significantly smaller Weber fraction than transcutaneous stimulation (blue) (*p* < 0.05, unpaired t-test).

Participants received transcutaneous stimulation through two 716-mm^2^ (30 mm diameter) adhesive electrodes (Series 530, Cardinal Health, Dublin, Ireland) placed on the anterior side of the wrist and electrocutaneous stimulation through a custom dry stimulation pad placed on the palm of the hand (Fig. 3A). The custom dry stimulation pad used for electrocutaneous stimulation was a 9 cm^2^ square silicone pad, 4 mm thick, and consisted of one 79 mm^2^ (10 mm diameter) stimulating electrode embedded in the center of the silicone surrounded by four 44 mm^2^ (7.5 mm diameter) ground electrodes also embedded in the silicone^8^. The electrode location for transcutaneous stimulation was chosen such that the electrical stimulation activated the sensory fibers of the *median* nerve, specifically the *palmar cutaneous branch*. The adhesive electrodes were chosen primarily based on their widespread availability and ability to maintain electrical connection over long durations of time and when the participant moves. The adhesive electrodes are designed for primary use as measurement electrodes, and do not have optimal characteristics for stimulation; however, they are widely available and have acceptable electrical properties to serve as stimulation electrodes for research purposes^15–17,35^. Both forms of stimulation produced an isolated paresthesia-like, pins-and-needles, tingling, prickling, or electrical percept on the palm of the hand. Stimulation consisted of biphasic, charge-balanced, square-wave, cathodic-first pulses, with 100-μs phase durations, and a 50-μs interphase duration. The pulse frequency varied between 12.5 and 200 Hz. Due to variations in skin impedance, the stimulation amplitude was chosen individually for each participant. Stimulation during experiments was performed at 1.5 mA above the detection threshold. The detection threshold is the lowest stimulation amplitude that a participant could feel stimulation for a given pulse width. If the maximum comfort level of stimulation amplitude was lower than 1.5 mA above the detection threshold, then the stimulation amplitude for that experiment was set to the maximum comfort level. The maximum comfort level was defined as the amplitude of stimulation that the participant would feel comfortable receiving continuously for an hour. Detection thresholds ranged from 1.7 to 2.5 mA for transcutaneous stimulation and from 1.2 to 1.6 mA for electrocutaneous stimulation.

We quantified the just-noticeable difference of changes in pulse frequency using a two-alternative forced-choice paradigm, as outlined in^8,43,44^. The participants were presented with two one-second stimulus trains separated by a one-second inter-stimulus interval. The participants were asked to respond to which of the two stimulus trains was more intense. The participants were allowed as much time as necessary to respond to the two-alternative forced-choice questions. Participants were instructed to ignore any changes in the quality, duration, or location of the percepts and to focus solely on the intensity or magnitude of the percept. Tactile stimuli, regardless of the difference in modality or quality, can all be judged on a single intensive continuum^45^.

The two stimulus trains were delivered at the same amplitude and pulse width but varied in pulse frequency. On each trial, one of the two stimulus trains served as the reference frequency and had a fixed pulse frequency of 50 Hz throughout the experiment. The second stimulus train served as a test frequency and had a pulse frequency that ranged from 25 to 175% of that of the reference frequency. For a given experiment, nine different test frequencies were explored, identical to those used in^8,44^. The order in which the test frequency and reference frequency appeared in a given trial was randomized. A total of 180 randomized trials were performed for the given reference frequency (i.e., 20 trials for each of the nine test frequencies).

Discrimination data at the nine test frequencies were fit with cumulative normal distributions to obtain psychometric functions. The just-noticeable difference (JND) was estimated as the change in pulse frequency that the participants could identify correctly 75% of the time. Each function provided two JNDs (one for decreases and one for increases in pulse frequency) which were averaged. To compare discriminability independent of the reference frequency, the Weber fraction was calculated by dividing the JND by the reference frequency.

All data were screened for normality and outliers prior to statistical analysis. All data were normally distributed. Outliers were defined as any value more than three scaled medians absolute deviations away from the dataset’s median and were removed from each metric. One of the participants was a statistical outlier and was removed from subsequent analysis. A one-sample unpaired t-test was used to compare the Weber fractions for transcutaneous (N = 6 participants) and electrocutaneous (N = 7 participants) stimulation. All bar plots and listed values are mean ± standard error of the mean.

### Transcutaneous motor stimulation

Eight healthy participants (1 male, 7 female; 21.88 ± 1.96 years; mean ± standard deviation) were recruited for this study. The participants had no known neurological or movement disorders. Informed consent and experimental protocols were carried out in accordance with the University of Utah Institutional Review Board (IRB 00110994).

Both nerve and muscle stimulation consisted of biphasic, charge-balanced, square-wave, cathodic-first pulses, with 200-μs phase durations, and a 50-μs interphase duration. The pulse frequency was set to 30 Hz. The stimulation amplitude varied from 0 to 13 mA based on the participant’s comfort and motor-twitch threshold.

A load cell (FX292X-100A-0100-L, TE Connectivity, Schaffhausen, Switzerland) with a custom 3D-printed casing was placed between the palm and the participant’s ring and middle fingers. The participants completed one experimental block consisting of 9 trials of squeezing the load cell with their maximum voluntary contraction (MVC) using only their ring and middle fingers against their palm. Each trial lasted for 7.5 s, followed by 2.5 s of rest before the next trial. Electrodes for nerve stimulation were placed on the inside of the upper arm between the biceps and triceps to activate the *median* and *ulnar* nerves, and electrodes for muscle stimulation were placed above the *flexor digiti profundus* in the forearm such that the ring and middle finger flexed when stimulated. The participants explored different stimulation amplitudes using a custom-built button interface until they decided on the maximum stimulus that they would be comfortable completing an experimental block with. The maximum stimulation for each stimulation site was then used to stimulate the participant for nine trials consisting of 7.5 s of contractions followed by 2.5 s of rest. The site that was stimulated first (muscle or nerve) and which arm was stimulated (left or right) were chosen randomly at the beginning of the experiment. Fifteen minutes of rest were given between experimental blocks to minimize fatigue. A custom LabVIEW (version 20.0.1, National Instruments, Austin, TX) interface and MATLAB (version 2020b, Mathworks Inc., Natick, MA) was used to set the stimulation parameters online and record data from the load cells.

The force measured by the load cell was recorded at 60 Hz. From the force traces, each trial’s maximum force, force onset time, and force rise time were measured. The force onset time was defined as the amount of time between the stimulus starting and the onset of force. The onset of force was determined as a force greater than 0.2 N to avoid false positives due to baseline sensor noise. Force rise time was defined as the length of time from force onset to 90% of the trial’s maximum evoked force. The fatigue rate from each experimental block was calculated using two methods, the first of which fit an exponential curve to the max forces of each experimental block normalized by the max force of the first trial^17^. The decay rates of the resulting exponential curves were then compared for significance. The second method of measuring fatigue consisted of dividing the max force of the last trial by the max force of the first trial. This is referred to as the percent force retained, similar to ^20^.

Outliers were defined as any value more than three scaled medians absolute deviations away from the dataset’s median and were removed from each metric. The resulting data was then verified to follow a normal distribution. Where no outliers were removed, a paired t-test was used to test significance. Otherwise, an unpaired t-test was used. All bar plots and listed values are the mean ± standard error of the mean.

### Ethics accordance

This study and all methods were approved by the University of Utah Institutional Review Board (IRB) (IRB study 00110994).

### Informed consent

Informed consent was obtained from all participants prior to the study.

## Results

### Verification of stimulator

The noninvasive stimulator was able to produce biphasic interleaved stimulation waveforms with varying pulse widths, pulse frequencies, and pulse amplitudes. Current steering is also possible through the simultaneous delivery of opposite-polarity monophasic pulses, or bipolar stimulation. The outputs of the noninvasive stimulator were connected to a 10 kΩ resistor such that 10 V on the oscilloscope corresponds to 1 mA of current (Fig. 2). Three interleaved pulses were generated across the three channels with the properly assigned currents (1, 2 and 3 mA), pulse widths (100, 200 and 300 µs), and frequencies (200 Hz) (Fig. 2B). The noninvasive stimulator was also able to produce bipolar waveforms (Fig. 2C). Bipolar stimulation can steer current between multiple stimulation sites and activate nerves that could not be activated using monopolar stimulation^1^. The loop speed of the microcontroller with three channels active was approximately 90 kHz (Table 1). An overshoot of negligible charge was sometimes observed when using a purely resistive load. This overshoot is typical of stimulators built around the Howland current source^40,41^. The rise time of the pulses was approximately 2 µs (Table 1), similar to rise times reported in^40–42^. The time resolution of the stimulator was 5 µs (Table 1).

The noninvasive stimulator was able to drive the current-controlled biphasic pulses across the skin (Fig. 2D). The previously described overshoot is not present when the electrodes are attached to the human body. No ringing was observed in the measurement of voltage across the skin.

### Sensory stimulation validation

Transcutaneous stimulation at the wrist and electrocutaneous stimulation on the palm both produced paresthesia-like percepts isolated to the palm (Fig. 3A). For both transcutaneous and electrocutaneous stimulation, participants discriminated changes in pulse frequency on the perceived intensity of the evoked percept. The just-noticeable difference (JND) for changes in pulse frequency at a 50-Hz reference frequency was 13.42 ± 3.98 Hz for transcutaneous stimulation and 6.51 ± 3.38 Hz for electrocutaneous stimulation. These just-noticeable differences equate to statistically different Weber fractions of 0.27 ± 0.08 for transcutaneous stimulation and 0.13 ± 0.04 for electrocutaneous stimulation (p < 0.003, unpaired t-test). The lower Weber fraction for electrocutaneous stimulation implies greater discriminability. Participants required a 27% increase in pulse frequency from the reference to reliably perceive a more intense transcutaneous stimulation, compared with a 13% increase in pulse frequency for electrocutaneous stimulation (Fig. 3B).

**Figure 4:**
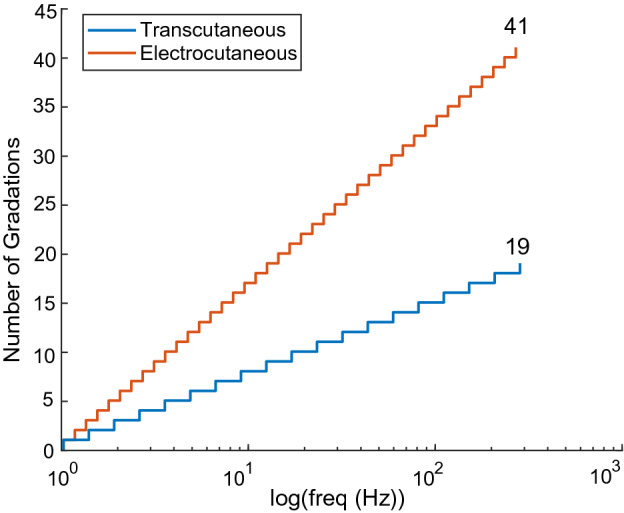
Transcutaneous (blue) has half of the number of sensory gradations than electrocutaneous (red) stimulation does.

Assuming a fixed range of stimulation pulse frequencies, the Weber fractions can be used to estimate the total number of perceivable sensory gradations possible. The total number of perceivable sensory gradations serves as a limit on the resolution for encoding the magnitude of tactile forces for assistive devices and virtual/augmented reality. We propose a fixed lower limit of stimulation at 1 Hz, below which closed-loop applications may be limited, and a fixed upper limit of stimulation at 300 Hz, as a conservative estimate of the maximum firing rate of cutaneous mechanoreceptors. When limiting pulse frequency between 1 to 300 Hz, electrocutaneous stimulation applied directly to the palm provides more than double the number of perceivable sensory gradations relative to that of transcutaneous stimulation applied via the wrist (Fig. 4). A total of 41 perceivable sensory gradations exists for electrocutaneous stimulation, whereas only 19 exist for transcutaneous stimulation.

### Motor stimulation validation

**Figure 5:**
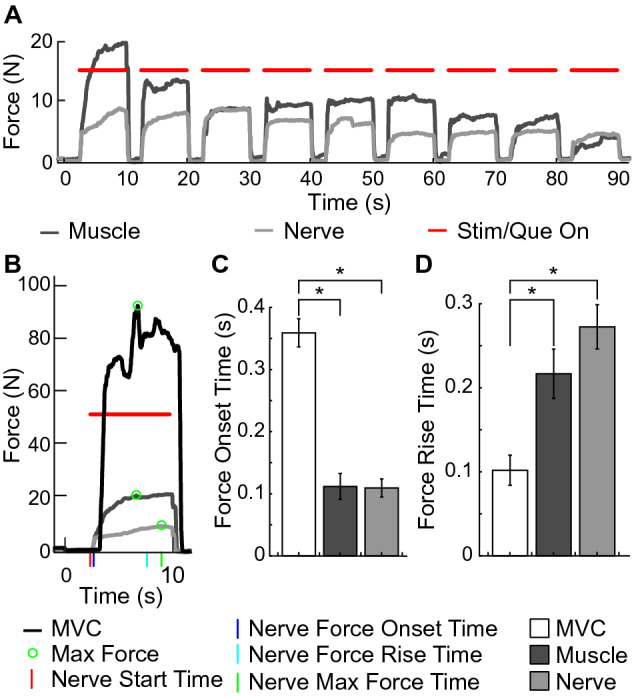
Overview of motor stimulation. (**A**) Example muscle (dark gray line) and nerve (light gray line) stimulation evoked grip force traces of a single participant. (**B**) A single trial with a corresponding maximum voluntary contraction (MVC) trace (black line). (**C**) Because of the participants’ response times to react to the stimuli, force onset time of MVC contractions (white bar; n = 70) was significantly slower than contractions evoked by muscle (dark gray bar; n = 69) and nerve (light gray bar; n = 64) stimulation (*p* < 0.05, unpaired t-test). (**D**) MVC contractions (n = 64) demonstrated a significantly faster force rise time than muscle (n = 67) or nerve (n = 68) stimulation evoked contractions (*p* < 0.05, unpaired t-test). No significant difference was found between the force onset time, or the force rise time of muscle and nerve stimulation evoked contractions. Bars show mean ± standard error of the mean. Asterisks denote statistical significance using the tests described above.

Stimulation at the muscle and nerve both produced reliable grip forces (Fig. 5A). Forces evoked by muscle and nerve stimulation had characteristic shapes resembling forces evoked by MVC, although they differed slightly in timing and amplitude (Fig. 5B). Muscle and nerve stimulation evoked forces with similar timings, while MVC had a slower force onset time and a faster force rise time.

Force onset time was defined as the time of the start of the trial to non-zero force output. As such, the force onset time was much greater for the MVC block due to the participants’ reaction times. Force onset time was 0.36 ± 0.02 s for MVC, 0.11 ± 0.02 s for muscle stimulation, and 0.11 ± 0.02 s for nerve stimulation (Fig. 5C). No significant difference was found between the force onset time of muscle stimulation and nerve stimulation (p = 0.93, unpaired t-test), but the force onset times of both stimulation methods were significantly different from the MVC force onset time (p’s < 0.05, unpaired t-tests).

The force rise time was 0.22 ± 0.03 s for muscle stimulation and 0.28 ± 0.03 s for nerve stimulation (Fig. 5D). No significant difference was found between the force rise time of muscle stimulation and of nerve stimulation (p = 0.16, unpaired t-test). The force rise time for MVC was significantly faster than both stimulation methods (p < 0.05, unpaired t-test).

**Figure 6:**
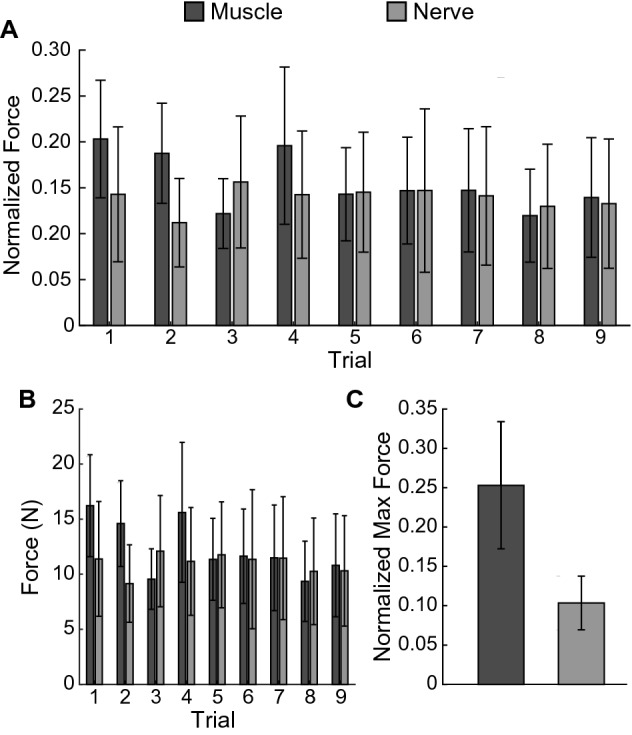
(**A**) The max force per trial normalized by each participant’s MVC and averaged across participants (*n* = 8) has similar magnitude throughout the experimental block for nerve (light gray bar) and muscle stimulation (dark gray bar). (**B**) The max forces of each trial were also similar magnitudes without normalization. (**C**) No significant difference was found between the maximum force evoked by muscle (n = 8) and nerve stimulation (n = 7), although the maximum force exerted by nerve stimulation tended to be much less than that exerted by muscle stimulation (*p* = 0.13, unpaired t-test). Bars show mean ± standard error of the mean.

Muscle forces evoked by muscle stimulation were initially greater than forces evoked by nerve stimulation, as noted by the maximum force of each trial normalized by the participant’s MVC (Fig. 6A) and without normalization (Fig. 6B). However, forces evoked by both muscle and nerve stimulation ultimately had similar forces by the ninth trial due to fatigue. The maximum grip force for muscle stimulation was 19.88 ± 5.88 N and 9.12 ± 3.17 N for nerve stimulation. No significant difference was found between the maximum grip force evoked during an experimental block by muscle and nerve stimulation (p = 0.15, unpaired t-test). When normalized by the participant’s MVC, the muscle stimulus maximum force was 0.25 ± 0.08 and the nerve stimulus maximum force was 0.10 ± 0.03 (Fig. 6C); again, no significant difference was found (p = 0.13, unpaired t-test).

**Figure 7:**
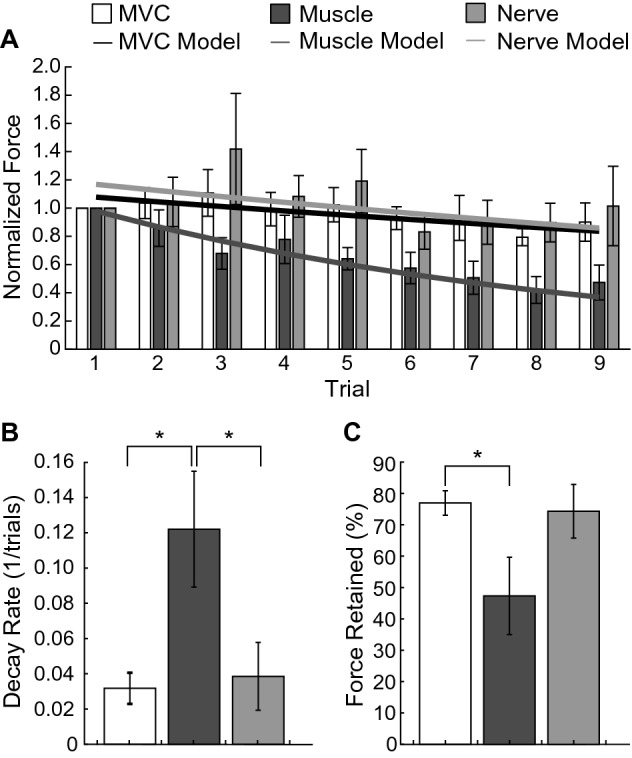
Nerve trunk stimulation resulted in a slower fatigue rate. (**A**) The bars indicate the maximum force of each trial normalized by the first trial of each experimental block, then averaged across participants, and the lines indicate the resulting exponential fatigue model fit. The black line represents the MVC fatigue model, the dark gray line represents the muscle fatigue model, and the light gray line represents the nerve fatigue model. (**B**) Muscle stimulation (dark gray bar; n = 8) resulted in a significantly faster exponential decay rate (paired t-test: *p* < 0.05) compared to nerve stimulation (light gray bar; n = 8) and MVC (white bar; n = 8). (**C**) Muscle stimulation (n = 7) also had a significantly lower force retention between the first and last trials (unpaired t-test: *p* = 0.05) than MVC (n = 8). Bars show mean ± standard error of the mean. Asterisks denote statistical significance using the tests described above.

When analyzing the fatigue associated with muscle and nerve stimulus, the maximum force of each trial was normalized by the maximum force of the experimental block’s first trial (Fig. 7A). Exponential models were fit to each participant’s normalized experimental block and then averaged across the participants. Directly stimulating the muscle resulted in faster fatigue rates than nerve stimulation. The decay rate of the evoked forces was 0.03 ± 0.01 trials-1 for MVC, 0.12 ± 0.03 trials-1 for muscle stimulus, and 0.04 ± 0.02 trials-1 for nerve stimulus (Fig. 7B). The force decay rate of the muscle stimulation block was significantly higher than the decay rate associated with the MVC block and nerve stimulation block (p < 0.05, paired t-test). No significant difference was found between the force decay rate of the MVC block and the nerve stimulation block (p = 0.68, paired t-test).

The percent force retained was 76.94 ± 3.91% for the MVC block, 47.35 ± 12.34% for the muscle stimulation block, and 74.30 ± 8.57% for the nerve stimulation block (Fig. 7C). The percent force retained for the muscle stimulation block was significantly lower than the MVC block (p = 0.05, unpaired t-test). No significant difference was found between the percent force retained of the MVC block and nerve block (p = 0.78, unpaired t-test) and the muscle and nerve block (p = 0.11, unpaired t-test).

## Discussion

The noninvasive stimulator introduced here provides a platform for a variety of different research studies using noninvasive neural stimulation. The noninvasive stimulator can drive biphasic, current-controlled stimulation through dry and gel electrodes to activate different neural structures, as validated with human subjects herein. The physical design is also open-source, inexpensive, portable, and can be controlled in real-time, enabling further use cases in research and education. Using this stimulator, we also performed some of the first-in-human comparisons of different neural targets for artificial sensory feedback and reanimating hand grasps.

### Electrocutaneous and transcutaneous stimulation for artificial sensory feedback from the hands

Using the noninvasive stimulator introduced here, we performed the first direct comparison of sensations evoked by noninvasive electrocutaneous stimulation and transcutaneous nerve stimulation and one of the first quantifications of the frequency discriminability of transcutaneous nerve stimulation. Electrocutaneous stimulation has been used extensively to evoke tactile sensations, often to provide sensory feedback to individuals suffering from limb loss^8,46–50^ or to improve the performance of human–machine interfaces such as virtual reality^51–53^. In contrast, transcutaneous nerve stimulation has only recently been explored for use in upper-limb prosthetics^54^ and virtual reality^55^. We build on these prior works by providing a direct comparison of the two approaches for artificial sensory feedback. We demonstrate that transcutaneous nerve stimulation applied at the wrist can evoke sensations in the hand, similar to the sensations evoked by electrocutaneous stimulation applied directly to the desired percept location. However, we show that electrocutaneous stimulation offers a greater ability to convey the magnitude of tactile sensations. That is, based on the Weber fractions reported here, electrocutaneous stimulation can theoretically produce a maximum of 41 distinctly perceivable levels of intensity, whereas transcutaneous stimulation can only produce a maximum of 19 distinctly perceivable levels of intensity.

Prior work has reported the Weber fractions for epineural stimulation and intraneural stimulation at 50 Hz to be 0.33^44^ and 0.10^8^, respectively. The Weber fraction associated with transcutaneous stimulation applied via the wrist at 50 Hz was 0.27, similar to 0.33 reported for epineural stimulation^44^ at 50 Hz. In contrast, electrocutaneous and intraneural stimulation had Weber fractions of 0.13 (our study) and 0.10^8^, respectively. These correlations in discriminability are possibly due to both transcutaneous and epineural stimulation targeting larger bundles of fibers compared to electrocutaneous and intraneural stimulation.

Despite the relatively limited discriminability, transcutaneous stimulation applied via the wrist offers a unique opportunity to provide artificial sensory feedback to the hand with perceptive fields distal to the site of stimulation. As such, artificial sensory feedback from the hand can be readily implemented in an elegant form factor such as a wristwatch or bracelet. Such a form factor would not cover the skin that contacts an object during grasp, thereby allowing for less inhibited sensory feedback in natural grasping. The ability to distally evoked percepts makes this design uniquely suited for augmented reality.

### Muscle and nerve stimulation for hand reanimation

The noninvasive stimulator introduced here was also used to provide a direct comparison of muscle stimulation and nerve stimulation for hand reanimation. Consistent with prior work, we show that both stimulation methods can reliably evoke muscle contractions^17,56,57^. Analysis of the individual trials indicated that muscle stimulation evoked larger forces at the beginning of an experimental block but decayed to less than the nerve stimulation forces by the end, resulting in higher fatigue rates for muscle stimulation. The exponential decay rate was 0.12 trial^-1^ for muscle stimulation forces and 0.04 trial^−1^ for nerve stimulation. Force decay rates in similar studies have found a decay rate of 0.02 s^−1^ for muscle stimulation and 12.1 × 10–3 s^−1^ for nerve stimulation^17^, corroborating the claim that nerve stimulation evokes fatigue-resistant muscle contractions.

No significant difference was found between the forces measured during muscle stimulation and nerve stimulation when the stimulation amplitudes were set according to participant comfort. The maximum forces were 19.88 ± 5.88 N and 9.12 ± 3.17 N for muscle and nerve stimulation, respectively. Similarly, another study compared muscle and nerve stimulation in the lower limb and also found no significant difference in force generation^56,57^.

In summary, we show that muscle stimulation and nerve stimulation generate similar forces with similar timings but that nerve stimulation does so with a slower fatigue rate. Since the forces and timings of muscle and nerve stimulation were not significantly different, it is likely that all control techniques developed for muscle functional electrical stimulation (FES) could be easily modified or directly applied to nerve FES. Nerve FES could serve as a fatigue-resistant form of reanimation in a rehabilitation setting.

While beyond the scope of this paper, it is important to note that nerve stimulation suffers from a long setup time dedicated to electrode placement due to a lack of distinguishable anatomy. Electrode placement on the muscle is straightforward as the muscle anatomy beneath the skin is well-defined. However, we found that the nerve stimulus site used to evoke similar movements in different participants varied in position and orientation, leading to large search times. Techniques like automated electrode mapping have the potential to significantly decrease the setup time^15,58^.

### Stimulator design

The noninvasive stimulator introduced here offers high compliance voltage on multiple customizable waveforms in a portable and inexpensive form factor. Each channel of the noninvasive stimulator consists of the bidirectional Howland current source presented in^34^ as the current driver and a digital potentiometer to control the stimulation amplitude. In this respect, our design is similar to that presented in^40^. In the present manuscript, we extend upon this prior work by further optimizing the circuitry, validating the design with human subjects for multiple neural targets, and making the design low-cost and open-source. Our design is unique in that it combines the waveform generation circuitry and stimulation driver into a single device that is controlled using a USB connection to a computer. Both the stimulation amplitude and shape (e.g., timing) are controlled by the microcontroller on which the stimulator circuitry is stacked on top of. Placing all the circuitry into a single, stackable form factor for a widely-used development platform makes the design inexpensive, compact, and easy to assemble. Other popular neuroscience educational tools use a similar form factor^38^.

Lastly, it’s important to emphasize that the presented noninvasive stimulator is not intended to be a medical device to treat medical conditions. Future work is required to ensure safety beyond supervised laboratory use approved by relevant human-subjects protection agencies.

## Conclusion

In this paper, a novel, portable, programmable, multichannel stimulator designed specifically for noninvasive stimulation was presented and validated on the benchtop and in human participants for sensory and motor stimulation. The design combined the waveform generation hardware with the current-driver circuitry to generate current-controlled biphasic waveforms across multiple channels. The stimulator offers real-time custom control of multiple waveforms and is portable, low-cost, and open-source, thereby enabling a variety of uses in research and education. Using the noninvasive stimulator presented here, we successfully activated and directly compared for the first time (a) afferent sensory fibers through electrocutaneous and transcutaneous stimulation and (b) motor fibers through direct muscle and nerve stimulation. As the field of neural engineering grows, low-cost, accessible, and customizable hardware can provide cutting-edge educational training and hands-on experience for the next generation of researchers.

## Supplementary Information

Supplementary Figure 1.

Supplementary Figure 2.

## Data Availability

The datasets used and/or analyzed during the current study available from the corresponding author on reasonable request.
